# Hematological characteristics, oxidative stress, and patient-reported symptoms in Tibetan patients with chronic mountain sickness at 4500 m altitude

**DOI:** 10.3389/fphys.2025.1661738

**Published:** 2025-09-11

**Authors:** Yang Zhong, Fengying Zhang, Qiuyue Li, Doudou Hao, Zhiyou Shi, Yuling Liu, Suying Zhu, Pasang Tsering, Yunhong Wu

**Affiliations:** ^1^ Tibet Autonomous Region Clinical Research Center for High-altitude Stress, Endocrinology and Metabolism Disease, Hospital of Chengdu Office of People’s Government of Xizang Autonomous Region (Hospital.C.X.), Chengdu, China; ^2^ Department of Biobank, Hospital of Chengdu Office of People’s Government of Xizang Autonomous Region (Hospital.C.X.), Chengdu, China; ^3^ Department of Endocrinology, Hospital of Chengdu Office of People’s Government of Xizang Autonomous Region (Hospital.C.X.), Chengdu, China; ^4^ Medicine College of Tibet University, Lhasa, China; ^5^ Research laboratory, Tibetan Hospital of Nagqu, Nagqu, China

**Keywords:** chronic mountain sickness, high-altitude polycythemia, oxidative stress, sleep quality, fatigue scale

## Abstract

**Background:**

Chronic mountain sickness (CMS), driven by chronic hypoxia, features erythrocytosis, cardiovascular impairment, and systemic oxidative stress. Current studies focus on haematological and cardiopulmonary changes, but multidimensional features like sleep disturbances, quality of life, and oxidative stress remain underexplored.

**Methods:**

The cross-sectional study included 47 adult Tibetan residents living at 4,500 m and diagnosed with CMS using Qinghai criteria. Blood samples were collected, and questionnaires assessed quality of life, fatigue, and sleep. Multivariate logistic regression was used to explore associations between variables, using CMS comorbid with high-altitude polycythemia (HAPC) or sleep disturbance as endpoints.

**Results:**

The mean age of patients was 40.57 ± 6.21 years (29 males, 18 females). Males had higher RBC, HGB, HCT, UA, and T-AOC levels (all *P* < 0.001). A moderate to strong positive correlation was observed between these markers. 91.67% of patients with comorbid HAPC were males with severe CMS. Lower MCHC (OR = 0.80, *P* = 0.02) and higher T-AOC (OR = 1.47, *P* = 0.02) were associated with HAPC. Males (OR = 0.11, *P* = 0.03), higher 8-OHdG levels (OR = 0.95, *P* = 0.03), higher body pain scores (OR = 0.91, *P* < 0.01), and higher general health scores (OR = 0.90, *P* = 0.02) were more likely to report good sleep quality.

**Conclusion:**

Males with CMS had higher T-AOC and better sleep quality than females. Good sleep quality was associated with better quality of life and less fatigue. Oxidative stress indicators correlated with clinical phenotypes, but causality requires further investigation. This trial was registered at Chinese Clinical Trial Registry (ChiCTR2400082685).

## Introduction

Millions of people travel to high-altitude regions annually, and over 80 million people live permanently above 2,500 m (Tremblay and Ainslie, 2021). Chronic exposure to high-altitude hypoxia can cause chronic mountain sickness (CMS), clinically characterised by excessive erythrocytosis, hypoxic pulmonary hypertension and multisystem dysfunction (Gatterer et al., 2024). The prevalence of CMS ranges from 5% to 33% across populations, with the highest rates documented in the Andean region (León-Velarde et al., 2005), and varies with altitude, age and genetic factors. Patients with CMS experience impaired quality of life and increased morbidity and mortality, attributable to marked arterial hypoxaemia and haemodynamic abnormalities. These haemodynamic abnormalities may drive the progression of pulmonary hypertension, cor pulmonale, left ventricular dysfunction, and thromboembolic complications (Swenson, 2022). Despite having evolved unique hypoxic adaptation mechanisms through genetic selection (Liu et al., 2020; Buroker et al., 2012) (e.g., variants in EPAS1 and EGLN1), Tibetan populations exhibit inter-individual susceptibility to CMS, reflecting a complex balance between adaptive compensation and pathophysiological dysregulation (Hsieh et al., 2016).

The pathological mechanisms of CMS are fundamentally linked to hypoxia-induced multisystem maladaptation. Studies demonstrate that both acute and chronic hypoxia elevate levels of oxidative stress biomarkers (Jefferson et al., 2004). Comparative analyses of high-altitude populations show significantly elevated levels of markers of oxidative stress (e.g., ascorbate free radicals) in CMS patients compared with non-CMS high-altitude residents (Bailey et al., 2013). Bailey et al. have further elucidated systemic oxidative stress (OS) characteristics in high-altitude populations, manifested through an imbalance between free radical generation and antioxidant defense mechanisms that sustains chronic oxidative stress. This pathological state interacts synergistically with inflammatory mediators, potentially accelerating cognitive deterioration and elevating depression susceptibility (Shanjun et al., 2020; Kong et al., 2011). In addition, high-altitude populations exhibit a significantly higher prevalence of sleep-disordered breathing (SDB) compared to sea-level populations. Recent studies have demonstrated that Andean high-altitude residents show a two-fold higher in the apnea-hypopnea index (AHI) compared to sea-level counterparts, primarily manifested by increased central sleep apnea events (Pham et al., 2017). Notably, the study by Ana Sanchez-Azofra’s team revealed no significant association between the progression of CMS and alterations in sleep architecture or the severity of SDB (Sanchez-Azofra et al., 2022). While these findings have significantly advanced our understanding of hypoxia-driven oxidative stress and sleep-related pathophysiology in CMS, the complex interplay remains underexplored.

Current research predominantly focuses on traditional domains including hypoxic erythrocytosis (Haase, 2013; Yang et al., 2024), pulmonary hypertension (Ye et al., 2023; El Alam et al., 2022; Naeije, 2019), and cardiovascular complications (Savina et al., 2024; Abondio et al., 2024), whereas systematic investigations into multidimensional characteristics of CMS patients - particularly oxidative stress regulatory mechanisms, sleep disorder phenotypes, fatigue severity, and quality of life - remain substantially understudied. There is an imperative need for enhanced clinical evidence to advance the pathophysiological understanding framework.

Therefore, we conducted a study screening Tibetan permanent residents living at 4,500 m for chronic mountain sickness (CMS). We systematically collected data on haematological parameters, blood biochemical profiles, oxidative stress biomarkers, and multidimensional clinical phenotypes (including sleep quality, fatigue level, and quality of life scores). By assessing correlations between these parameters, we aimed to elucidate the pathophysiological features of CMS and to establish a scientific basis for developing prevention and treatment strategies.

## Materials and methods

### Participants

The study recruited 47 Tibetan patients with chronic mountain sickness (CMS) from Nagqu City (altitude 4,500 m), Tibet Autonomous Region, using convenience sampling. Inclusion criteria were: (1) Tibetan ethnicity; (2) aged 18–60 years; (3) body mass index (BMI) 18–28 kg/cm^2^; (4) permanent residents (residing locally for ≥6 months in the past year); (5) meeting the Qinghai diagnostic criteria for CMS (León-Velarde et al., 2005); and (6) non-smokers and non-drinker. Exclusion criteria were: (1) polycythemia vera or secondary polycythemia; (2) organic sleep disorders; (3) pregnancy or lactation; and (4) severe hepatic or renal dysfunction. The study protocol was approved by the Ethics Review Committee of the Hospital of Chengdu Office of People’s Government of Xizang Autonomous Region (2024-EC-073). The clinical trial was registered at the China Clinical Trial Registry (ChiCTR2400082685). The design and data collection for this study were based on the baseline survey conducted within that registered trial. All participants provided written informed consent.

### Measures

#### Blood biochemistry and physical measurements

Following an overnight fasting period, venous blood samples were collected from all participants between 08:00 and 10:00. Whole blood was drawn into EDTA-coated tubes for complete blood count (CBC) analysis (BC-6100 automated hematology analyser, Mindray, Shenzhen, China), including: red blood cell count (RBC, 10^12^/L), haemoglobin concentration (HGB, g/L), haematocrit (HCT, %), mean corpuscular volume (MCV, fL), mean corpuscular haemoglobin (MCH, pg), mean corpuscular haemoglobin concentration (MCHC, g/L), red blood cell distribution width - coefficient of variation (RDW-CV, %), red blood cell distribution width - standard deviation (RDW-SD, fL), platelet count (PLT, 10^9^/L), mean platelet volume (MPV, fL), and platelet distribution width (PDW, fL). Blood was also drawn into serum-separating tubes for biochemical profiling (BS-800 M automated biochemistry analyser, Mindray, Shenzhen, China). Serum was obtained by centrifugation (3,000 g for 15 min at 4 °C) and stored at −80 °C until analysis. Biochemical assays included: total cholesterol (CHOL, mmol/L), triglycerides (TG, µmol/L), high-density lipoprotein cholesterol (HDL-C, µmol/L), low-density lipoprotein cholesterol (LDL-C, µmol/L), apolipoprotein A1 (ApoA1, g/L), apolipoprotein B (ApoB, g/L), blood urea nitrogen (BUN, mmol/L), creatinine (Cr, mmol/L), uric acid (UA, µmol/L), and glucose (GLU, mmol/L).

Trained staff conducted anthropometric measurements using calibrated instruments: standing height and weight were measured with a stadiometer and digital scale (IPR-scale 02), from which body mass index (BMI) was calculated. Blood pressure was measured using an automated sphygmomanometer (Omron HEM-1000) after participants had rested in the seated position for 5 min, with triplicate measurements taken at 2-min intervals.

#### Oxidative stress biomarkers

Oxidative stress was measured by centrifuging 2 mL of whole blood (instruments used for sample pretreatment are detailed in the Supplementary Table S6) and storing the serum at −80 °C until the samples were tested. All colorimetric assays were performed using a μQuant microplate spectrophotometer (BioTek, Winooski, VT, United States). Reduced glutathione (GSH, umol/L) was estimated using GSH colorimetric assay kit (E-BC-K030-M, Elabscience, Houston, TX, United States) according to the method described by Beutler et al. (1963), with absorbance measured at 405 nm. Lipid peroxidation was estimated using a malondialdehyde (MDA, umol/L) colorimetric assay kit (E-BC-K025-M, Elabscience, Houston, TX, United States) by measuring thiobarbituric acid reactive substance (TBARS) and expressed in terms of MDA content according to Ohkawa et al. (1979). MDA, the final product of fatty acid peroxidation, reacts with thiobarbituric acid (TBA) to form a colored complex, the absorbance of which was measured at 532 nm in the supernatant. Superoxide dismutase (SOD, umol/L) activity using SOD typed activity assay kit (E-BC-K020-M, Elabscience, Houston, TX, United States) was determined according to Giannopolitis and Ries (Giannopolitis and Ries, 1977). The color reaction was measured at 550 nm. Catalase (CAT, U/mL) activity was determined using a CAT activity assay kit (E-BC-K031-M, Elabscience, Houston, TX, United States) according to the method of Aebi (Aebi, 1984). Total antioxidant capacity (T-AOC, U/mL) was measured using a colorimetric assay kit (E-BC-K136-M, Elabscience, Houston, TX, United States), and one unit of total antioxidant capacity was assigned for each 0.01 increase in the absorbance of the reaction system per millilitre of sample per minute at 37 °C. 8-OHdG was determined using an ELISA technique (E-EL-0028, Elabscience, Houston, TX, United States) performed according to the kit instructions and the absorbance values were measured at 450 nm using an enzyme marker and the results were expressed in ng/mL.

#### Clinical phenotypes

The Pittsburgh Sleep Quality Index (PSQI) is a widely used and validated questionnaire for assessing sleep quality across diverse populations (Buysse et al., 1989; Cole et al., 2006). As a subjective measure, the PSQI evaluates self-reported sleep quality and disturbances over a 1-month period. In this study, the 19 self-rated items of the PSQI were combined into seven component scores: subjective sleep quality, sleep latency, sleep duration, habitual sleep efficiency, sleep disturbances, use of sleep medication, and daytime dysfunction (Buysse et al., 1989). Each component is scored from 0 to 3 (except subjective sleep quality, which is scored from 1 to 3), with higher scores indicating poorer sleep outcomes. The global PSQI score, calculated by summing all seven components, provides a comprehensive measure of overall sleep quality. For Chinese populations, a global PSQI score >7 identifies individuals with poor sleep quality, with a sensitivity of 98.3% and a specificity of 90.2% (Liu, 1996).

The fatigue severity was assessed using the FS-14, a standardised 14-item questionnaire. Each item provides a binary response (Yes/No), scored as 0 or 1, yielding total scores ranging from 0 to 14 (Chalder et al., 1993; Jing et al., 2016). Higher total scores indicate greater severity of chronic fatigue.

Health-related quality of life (HRQoL) was assessed using the SF-36® questionnaire, a widely utilized instrument with demonstrated validity, reliability, and appropriateness for HRQoL measurement (Brazier et al., 1992; McHorney et al., 1994; Ware, 2000). The SF-36® score comprises eight domains: physical functioning (PF), role limitations due to physical problems (RP), bodily pain (BP), vitality (VT), general health perceptions (GH), mental health (MH), social functioning (SF), and role limitations due to emotional problems (RE). Each domain score ranges from 0 to 100, with lower scores indicating greater severity of physical or mental disability.

Professionally trained investigators (proficient in both Tibetan and Chinese) administered all questionnaires to participants via structured interviews, using the official Tibetan language versions. The scoring criteria for each scale were detailed in Supplementary Table S7. The entire data collection process underwent audio-recorded quality control, with random verification checks conducted daily post-survey to ensure data integrity.

### Sample size

The required sample size was calculated based on reports in the literature that CMS patients typically exhibit HGB levels >210 g/L in males and >190 g/L in females. Assuming a standard deviation (SD) of 20 g/L, an allowable error) of 10 g/L, a significance level (α) of 0.05, and a power (1-β) of 0.80, the minimum required sample size was estimated as 34 participants using PASS software (version 15.0; Power Analysis and Sample Size). To account for an anticipated 20% data loss rate, the final sample size was set at a minimum of 43 participants.

### Statistical analysis

For continuous variables, we assessed normality using the Shapiro-Wilk test. Based on the normality test results, data are presented as mean ± standard deviation or median (interquartile range), as appropriate. Between-group comparisons were made using the Student’s t-test or the Wilcoxon rank-sum test. Categorical variables are presented as number (%) and compared using the χ^2^ test or Fisher’s exact test, as appropriate. Correlation analysis was performed using Pearson’s or Spearman’s correlation coefficient, depending on the distribution of the variables. To identify independent associations, we constructed multivariable logistic regression models. The outcome variables were the presence of high-altitude polycythemia (HAPC) or the presence of a sleep disorder (defined as PSQI >7). Variables for inclusion in the final models were selected using a stepwise selection method (forward and backward), with a significance level of P < 0.05 for entry and P > 0.10 for removal.

All analyses were performed using R software (version 4.4.3). A *P* value of <0.05 was considered statistically significant.

## Results

### Characteristics of the sample population

The 47 patients with CMS had a mean age of 40.57 ± 6.21 years and a mean BMI of 25.10 ± 2.32 kg/m^2^, comprising 29 males (61.70%) and 18 females (38.30%). Forty-five patients (95.74%) had moderate to severe CMS (Table 1). The proportion of severe CMS was significantly higher in males than females (68.97% vs 27.78%). Males also had significantly higher levels of RBC, HGB, HCT, PDW, CHOL, TG, LDLc, and UA (all *P* < 0.05). Among oxidative stress markers, T-AOC levels were significantly higher in males than females (17.67 ± 4.71 U/mL vs 10.10 ± 5.31 U/mL, *P* < 0.001). No significant differences were found between males and females in fatigue or quality of life scores, except for sleep quality (Supplementary Table S1).

**TABLE 1:** Basic characteristics of 47 Tibetan patients with chronic mountain sickness.

Variable	Total (N = 47)	Sex		*P*
		Males (n = 29)	Females (n = 18)	
Age, years	40.57 ± 6.21	41.86 ± 5.89	38.50 ± 6.32	0.078
HAPC				0.017
Yes	12 (25.53)	11 (37.93)	1 (5.56)	
No	35 (74.47)	18 (62.07)	17 (94.44)	
CMS				0.012
Mild	2 (4.26)	1 (3.45)	1 (5.56)	
Moderate	20 (42.55)	8 (27.59)	12 (66.67)	
Severe	25 (53.19)	20 (68.97)	5 (27.78)	
BMI, kg/m^2^	25.10 ± 2.32	25.55 ± 2.25	24.37 ± 2.30	0.094
SBP, mmHg	116.00 (106.00, 122.50)	118.00 (109.00, 128.00)	110.00 (106.00, 116.75)	0.120
DBP, mmHg	78.36 ± 10.91	79.34 ± 11.23	76.78 ± 10.49	0.432
RBC, 10^12/L	6.27 (5.65, 6.91)	6.64 (6.27, 7.77)	5.58 (5.20, 5.85)	<0.001
HGB, g/L	189.77 ± 32.02	207.69 ± 25.30	160.89 ± 17.00	<0.001
HCT, %	55.60 (50.65, 61.25)	59.50 (56.00, 70.90)	48.10 (45.35, 51.08)	<0.001
MCV, fL	88.79 ± 4.82	89.64 ± 4.63	87.42 ± 4.93	0.133
MCH, pg	29.60 (28.55, 31.00)	30.10 (29.10, 31.00)	28.70 (28.40, 30.78)	0.158
MCHC, g/L	334.00 (329.00, 338.00)	334.00 (329.00, 339.00)	333.00 (328.25, 337.75)	0.554
RDWcv, %	13.90 (13.40, 14.55)	13.80 (13.40, 14.40)	13.90 (13.10, 15.17)	0.93
RDWsd, fL	46.90 (45.50, 49.10)	47.60 (45.70, 49.30)	46.50 (45.25, 48.10)	0.255
PLT, 10^9/L	237.81 ± 71.75	223.83 ± 66.07	260.33 ± 76.61	0.104
MPV, fL	10.37 ± 1.07	10.31 ± 1.02	10.47 ± 1.16	0.622
PDW, fL	16.31 ± 0.39	16.46 ± 0.34	16.09 ± 0.36	0.002
CHOL, mmol/L	3.95 (3.50, 4.58)	4.16 (3.80, 4.78)	3.52 (3.27, 4.11)	0.014
TG, umol/L	0.89 (0.65, 1.36)	1.23 (0.85, 1.46)	0.61 (0.54, 0.84)	<0.001
HDLc, umol/L	1.24 (0.92, 1.49)	1.21 (0.92, 1.49)	1.31 (0.92, 1.48)	0.431
LDLc, umol/L	3.12 (2.60, 3.62)	3.39 (2.94, 4.01)	2.62 (2.13, 3.08)	0.001
ApoA1, g/L	1.49 ± 0.18	1.45 ± 0.14	1.56 ± 0.21	0.063
ApoB, g/L	0.92 ± 0.31	1.02 ± 0.31	0.75 ± 0.25	0.002
BUN, mmol/L	4.72 ± 1.50	5.16 ± 1.55	4.01 ± 1.14	0.006
Cr, mmol/L	77.76 ± 12.87	83.56 ± 8.78	68.42 ± 13.09	<0.001
UA, umol/L	387.78 ± 115.19	436.72 ± 110.31	308.94 ± 72.09	<0.001
GLU, mmol/L	4.62 ± 0.59	4.61 ± 0.64	4.65 ± 0.51	0.773
T-AOC, U/mL	14.77 ± 6.15	17.67 ± 4.71	10.10 ± 5.31	<0.001
CAT, U/mL	120.80 (87.66, 171.82)	127.07 (82.87, 182.60)	118.79 (90.23, 142.40)	0.753
GSH, umol/L	22.48 (20.17, 29.68)	23.05 (20.75, 29.39)	21.90 (18.88, 29.54)	0.751
MDA, umol/L	3.54 ± 1.54	3.82 ± 1.71	3.09 ± 1.11	0.085
SOD, umol/L	48.04 (45.80, 52.08)	47.75 (45.21, 50.59)	49.46 (47.90, 54.10)	0.120
8-OHdG, ng/mL	23.03 (17.38, 37.35)	24.59 (17.96, 36.53)	22.61 (14.81, 37.44)	0.686

Data were expressed as mean ± standard deviation, median (quartiles), or n (percentage). Comparisons between groups were made using t-test or Wilcoxon test. Abbreviations: HAPC (high altitude polycythemia; Yes/No), CMS (chronic mountain sickness; Mild/Moderate/Severe), BMI (body mass index), SBP/DBP (systolic/diastolic blood pressure), RBC (red blood cells), HGB (hemoglobin), HCT (hematocrit), MCV (mean corpuscular volume), MCH (mean corpuscular hemoglobin), MCHC (MCH, concentration), RDWcv/RDWsd (red cell distribution width, coefficient of variation/standard deviation), PLT (platelet count), MPV (mean platelet volume), PDW (platelet distribution width), CHOL (total cholesterol), TG (triglycerides), HDLc/LDLc (high/low-density lipoprotein cholesterol), ApoA1/ApoB (apolipoprotein A1/B), BUN (blood urea nitrogen), Cr (creatinine), UA (uric acid), GLU (glucose), T-AOC (total antioxidant capacity), CAT (catalase), GSH (glutathione), MDA (malondialdehyde), SOD (superoxide dismutase), and 8-OHdG (8-hydroxy-2′-deoxyguanosine).

### Correlation analysis of measured parameters

Among haematological parameters, RBC, HGB and HCT demonstrated moderate positive correlations with UA (*r* = 0.55, 0.55 and 0.54, respectively; all *P* < 0.001). These parameters also showed strong positive correlations with T-AOC (*r* = 0.66, 0.75, and 0.72, respectively; all *P* < 0.001).

**FIGURE 1:**
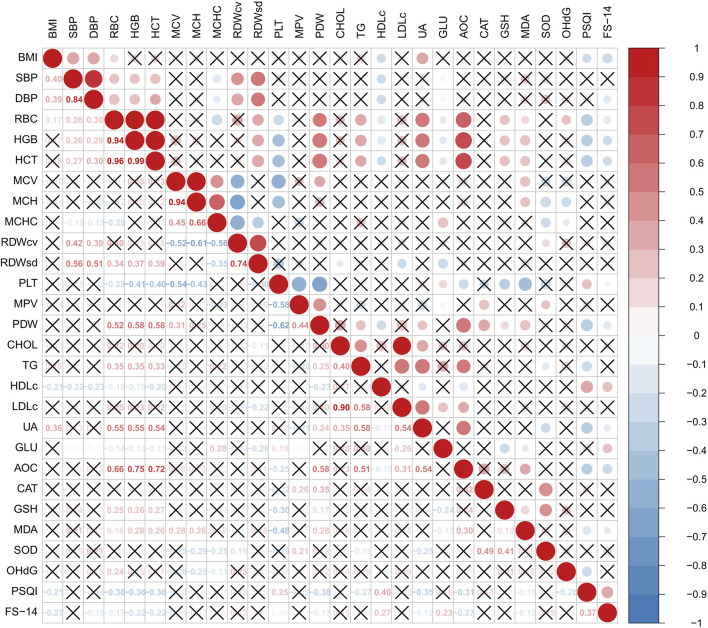
Correlation analysis of measured parameters.

The PSQI index was negatively correlated with RBC, HGB, HCT, PDW, UA and T-AOC, and positively correlated with HDLc and ApoA1 (all *P* < 0.05, Figure 1).

### Factors associated with high altitude polycythemia

**FIGURE 2:**
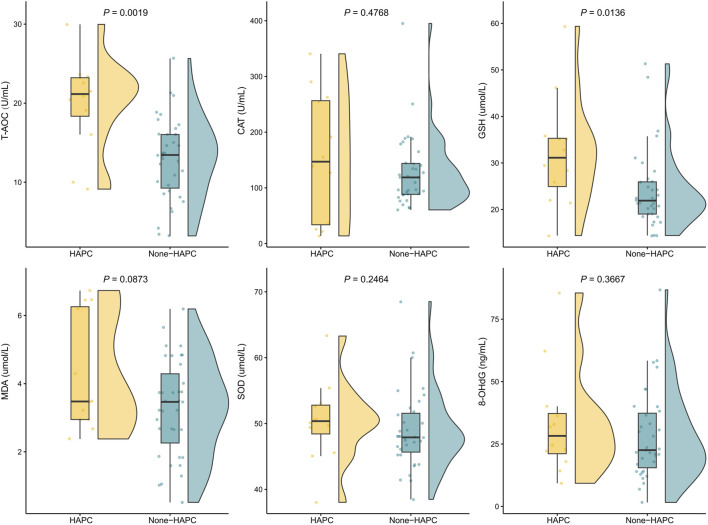
Differences between the groups (HAPC vs non-HAPC) in indicators of oxidative stress. Comparisons between groups were made using t-test or Wilcoxon test.

Among the 47 patients with chronic altitude sickness, 12 had HAPC. Eleven (91.67%) of these 12 patients were male and had severe chronic altitude sickness. Both systolic blood pressure (SBP) and diastolic blood pressure (DBP) were higher in patients with HAPC compared to those without HAPC (*P* < 0.05, Supplementary Table S2). Levels of T-AOC and GSH were also higher in patients with HAPC compared to those without HAPC (all *P* < 0.05, Figure 2). Other measures of sleep quality, fatigue and quality of life showed no statistically significant differences (Supplementary Table S3).

Multivariate analysis showed that after adjustment for sex, MCHC and RDWsd, showed that higher T-AOC levels were positively associated with the risk of HAPC (OR = 1.47, 95% CI: 1.06–2.04, Table 2).

**TABLE 2:** Multivariate logistic regression analysis of factors associated with HAPC in Tibetan patients with chronic mountain sickness.

Variable	*β*	*SE*	*Wald X* ^*2*^	*P*	*OR (95%CI)*
Intercept	51.98	32.21	2.6	0.1066	
Sex					
Males	0.84	1.79	0.22	0.6413	2.31 (0.07,77.64)
Females					1
MCHC, g/L	−0.23	0.09	5.8	0.016	0.80 (0.66, 0.96)
RDWsd, fL	0.31	0.21	2.05	0.1525	1.36 (0.89, 2.06)
T-AOC, U/mL	0.38	0.17	5.25	0.0219	1.47 (1.06, 2.04)

No-HAPC, as reference. Abbreviations: MCHC (mean corpuscular hemoglobin concentration), RDWsd (red cell distribution width standard deviation), and T-AOC (total antioxidant capacity).

### Factors associated with sleep quality

**FIGURE 3:**
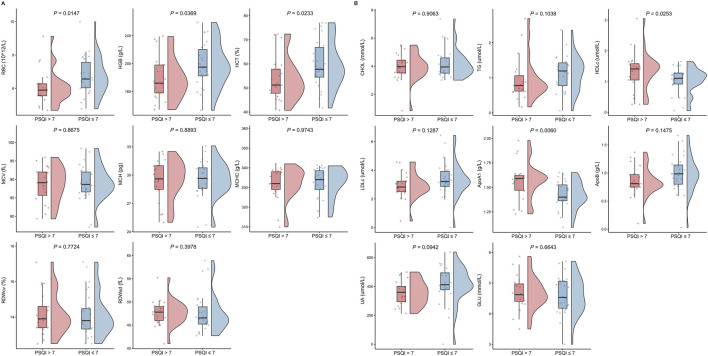
Differences in CBC parameters between groups (PSQI >7 vs PSQI ≤7). Comparisons between groups were made using t-test or Wilcoxon test.

**FIGURE 4:**
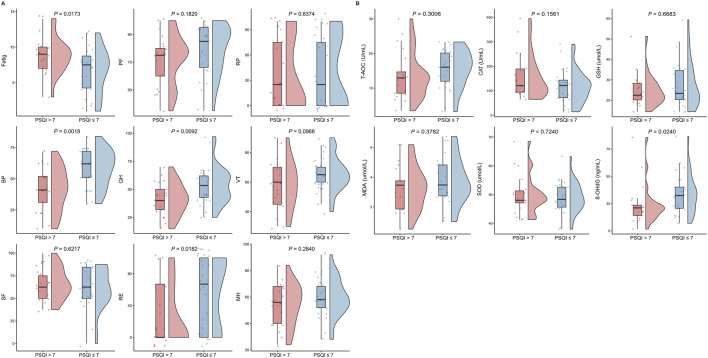
Differences in fatigue scales, SF-36 scales, and oxidative stress indicators between groups (PSQI >7 vs PSQI ≤7). Comparisons between groups were made using t-test or Wilcoxon test.

In the overall study population, individuals with good sleep quality had higher RBC, HGB and HCT levels and lower HDLc and ApoA1 levels compared to those with sleep problems (all *P* < 0.05, Figure 3). Levels of 8-OHdG were significantly elevated in the group with good sleep quality (*P* = 0.024, Supplementary Table S4). These subjects also exhibited lower fatigue scores and higher BP, GH and RE scores (all *P* < 0.05, Figure 4; Supplementary Table S5).

In multivariate logistic regression analysis (Table 3), male subjects was significantly associated with lower odds of poor sleep quality (OR = 0.11, 95% CI: 0.01–0.81). Furthermore, each 1-unit increase in 8-OHdG was associated with a 5% reduction in the odds of poor sleep quality (OR = 0.95, 95% CI: 0.90–0.99). Similarly, better PSQI scores for BP (OR = 0.91, 95% CI: 0.85–0.97) and GH (OR = 0.90, 95% CI: 0.83–0.98) were significantly associated with lower odds of poor sleep quality.

**TABLE 3:** Multivariate logistic regression analysis of factors associated with sleep quality in Tibetan patients with chronic mountain sickness.

Variable	*β*	*SE*	*Wald X* ^*2*^	*P*	*OR (95%CI)*
Intercept	12.3	3.82	10.34	0.001	
Sex					
Males	−2.25	1.04	4.67	0.031	0.11 (0.01,0.81)
Females					1
8-OHdG, ng/mL	−0.06	0.03	4.98	0.026	0.95 (0.90, 0.99)
BP	−0.09	0.03	7.32	0.007	0.91 (0.85, 0.97)
GH	−0.1	0.04	5.84	0.016	0.90 (0.83, 0.98)

PSQI <7 as reference. Abbreviations: 8-OHdG (8-hydroxy-2′-deoxyguanosine), BP (bodily pain), and GH (general health perceptions).

## Discussion

CMS remains a significant health challenge for permanent residents at high altitudes. Current research on CMS at extreme altitudes (>4500 m) remains limited, predominantly focusing on migrant populations and males cohorts (Jiang et al., 2014; Champigneulle et al., 2022; Oberholzer et al., 2020). This study assessed long-term Tibetan residents (both sexes) living at 4500 m, analysing their oxidative stress, sleep quality, fatigue and quality of life. Key findings revealed that male CMS patients exhibited significantly higher RBC, HGB, HCT, UA and T-AOC compared with females. Analysis of the total population demonstrated significant positive correlations between RBC, HGB, HCT and T-AOC. Notably, 91.67% of CMS patients with HAPC were male and presented with severe CMS. Multivariate analysis identified two independent factors associated with HAPC comorbidity: decreased MCHC (OR = 0.80) and increased T-AOC (OR = 1.47). Furthermore, better sleep quality was significantly associated with higher 8-OHdG (OR = 0.95), and better scores on the bodily pain (BP) (OR = 0.91) and general health (GH) (OR = 0.90) domains.

Oxidative stress is implicated in the pathogenesis of several human diseases, while antioxidants regulate redox homeostasis and signalling pathways. OS occurs when endogenous antioxidant defences are overwhelmed, leading to molecular, tissue and cellular damage (Silvestrini and Mancini, 2024). T-AOC is a biomarker measuring the antioxidant potential of body fluids (Manafikhi et al., 2017). In this study, we observed positive correlations between HGB, UA, and T-AOC in CMS patients, with HGB, UA and T-AOC levels being higher in males than in females. Epidemiological studies indicated that humans at high altitudes, characterised by low pressure and low oxygen, experience elevated HGB and UA levels (Pu et al., 2024). Previous experimental studies demonstrated that uric acid influences T-AOC measurements by direct scavenging of free radicals, synergistic enhancing other antioxidant systems, and elevating of humoral antioxidant reserves (Cao and Prior, 1998). Consequently, the endogenous antioxidant system may exhibit responsiveness to oxidative stress during hypoxia. Uric acid, as an endogenous antioxidant, may play an important protective role against oxidative stress in the context of systemic hypoxia during high-altitude exposure, particularly when blood viscosity is increased (Baillie et al., 2007).

In patients with CMS complicated by HAPC, elevated levels of antioxidants (including T-AOC and GSH) were observed compared to those with CMS alone. This paradoxical phenomenon, characterized by an enhanced antioxidant response correlating with greater disease severity, contrasts with previous reports. For example, a study using chronic hypobaric hypoxia-induced CMS rat models (simulated 5,000 m altitude for 30 days) showed that the CMS group had significantly elevated MDA levels and decreased SOD and GSH levels compared to the normal control group (Ma et al., 2014). Additionally, Bailey et al. reported that CMS patients at high altitude had lower levels of GSH and higher levels of oxidised glutathione (GSSG) than non-CMS individuals, and that these oxidative and inflammatory responses were associated with cognitive decline and depressive symptoms (Bailey et al., 2019). In this study, HGB influenced CMS disease severity and was positively correlated with UA and T-AOC, which may account for the observed higher antioxidant levels in more severe CMS. Future studies could investigate the relationship between CMS disease severity and antioxidant levels after controlling for HGB.

The present study observed that CMS patients with good sleep quality exhibited lower levels of bodily pain, improved general health perceptions and reduced fatigue indices, suggesting a potentially optimal adaptive state. Notably, however, patients with good sleep quality predominated in males and had significantly higher levels of HGB and the oxidative stress marker 8-OHdG. Both the greater propensity of males to self-report favourable health status compared to females (Meyer et al., 2014) and a possible association between HGB concentrations and sleep quality parameters may explain the observed males predominance and elevated HGB levels. Sanchez-Azofra et al. demonstrated that isovolemic hemodilution in patients with CMS resulted in decreased HGB levels, but acutely exacerbated nocturnal oxygen saturation and aggravated the severity of sleep apnea (Sanchez-Azofra et al., 2022). 8-OHdG, a biomarker of RNA or DNA oxidation, is widely used in disease mechanism research, environmental exposure assessment and drug efficacy monitoring. Its levels are influenced by gender, age, smoking and physical activity (Peres et al., 2020). On the one hand, studies have shown that 8-OHdG is associated with a variety of disease risks. Specifically, higher 8-OHdG levels show a positive association with cardiovascular disease (CVD) incidence in females, but a U-shaped association in males (Nagao et al., 2020). Elevated plasma 8-OHdG is associated with increased motor cognitive risk (MCR) in older adults, and Alzheimer’s disease patients exhibit higher urinary 8-OHdG levels than healthy elderly controls (Dai et al., 2024; Zengi et al., 2012). On the other hand, previous studies have postulated that this phenomenon may be related to inter-individual variations in DNA damage repair capacity. When exposed to comparable levels of oxidative damage, individuals with enhanced repair functionality may demonstrate more efficient excision and subsequent repair of 8-OHdG adducts on DNA strands, thereby exhibiting relatively elevated urinary excretion levels of this oxidative damage biomarker (Il’yasova et al., 2012). However, a prospective epidemiological study by Loft et al. found no significant association between lung cancer risk and urinary 8-OHdG excretion (Loft et al., 2006). Similarly, Peres et al. showed in their cross-sectional study that the severity of obstructive sleep apnoea (OSA) was not significantly associated with 8-OHdG biomarkers (Peres et al., 2020). Therefore, whether the higher blood 8-OHdG levels observed in CMS patients with good sleep quality reflect more severe DNA oxidative damage or enhanced DNA repair capacity needs to be further investigated in prospective studies.

Although this study has strengths, the findings should be interpreted considering several limitations. First, the absence of a representative control group (e.g., individuals without CMS matched for gender and age) limits the ability to directly compare biomarker levels between CMS and non-CMS populations. Second, convenience sampling method used in these high-altitude areas, characterised by low population density and dispersed distribution, may introduce selection bias, potentially limiting the generalizability of the findings. Third, the assessment of sleep quality using the PSQI questionnaire in CMS patients may be subject to participants’ subjective perceptions and cognitive biases. Fourth, as a small-scale cross-sectional study, this research can only provide evidence of associations rather than establish causal relationships. Further validation through prospective studies with larger sample sizes is warranted.

## Conclusion

In summary, this study reveals sex-specific characteristics in CMS patients, with males demonstrating elevated hematological indices (RBC, HGB, HCT) and T-AOC compared to females. Notably, HAPC comorbidity occurred primarily in severe male CMS cases and was associated with lower MCHC and higher AOC levels. Paradoxically, male sex and elevated oxidative stress markers (8-OHdG) coexisted with improved sleep quality, suggesting complex compensatory mechanisms in chronic hypoxic adaptation. These findings provide a scientific basis for understanding the haematological, oxidative stress and patient-reported characteristics of CMS patients at extreme altitudes.

## Data Availability

The datasets presented in this article are not readily available because All data underlying this study will be made available upon reasonable request by the corresponding authors. Requests to access the datasets should be directed to zyzhongyang1126@163.com.
