# Supplementary material for: Hematological characteristics, oxidative stress, and patient-reported symptoms in Tibetan patients with chronic mountain sickness at 4500 m altitude
Source: Front Physiol. 2025 Sep 11;16:1661738. doi: 10.3389/fphys.2025.1661738 (PMC12460463; doi:10.3389/fphys.2025.1661738)
Supplement: Supplementary file 1 [file Supplementaryfile1.docx]

**Supplementary file 1**

**Hematological characteristics, oxidative stress, and patient-reported symptoms in Tibetan patients with chronic mountain sickness at 4500m altitude**

**Yang Zhong^1,2#^, Fengying Zhang^3#^, Qiuyue Li^4^, Doudou Hao^2^, Zhiyou Shi^5^, Yuling Liu^5^, Suying Zhu^5^, Pasang Tsering^6*^, Yunhong Wu^1,4*^**

1. Tibet Autonomous Region Clinical Research Center for High-altitude Stress, Endocrinology and Metabolism Disease, Hospital of Chengdu Office of People's Government of Xizang Autonomous Region (Hospital.C.X.), Chengdu, China
2. Department of Biobank, Hospital of Chengdu Office of People's Government of Xizang Autonomous Region (Hospital.C.X.), Chengdu, China
3. Clinical Trial Center, National Medical Products Administration Key Laboratory for Clinical Research and Evaluation of Innovative Drugs, West China Hospital, Sichuan University, Chengdu, China
4. Department of Endocrinology, Hospital of Chengdu Office of People's Government of Xizang Autonomous Region (Hospital.C.X.), Chengdu 610041, China
5. Medicine College of Tibet University, Lhasa, China
6. Research laboratory, Tibetan Hospital of Nagqu, Naqu, China

# shared first authorship.

* shared corresponding authorship.

Corresponding author:

Yunhong Wu, Email: wu_yunhong@163.com

Table S1. Basic characteristics of 47 Tibetan patients with chronic altitude sickness

Table S2. Differences in basic indicators between Tibetan patients with HAPC and those without HAPC.

Table S3. Differences in sleep, fatigue, and quality of life between Tibetan patients with HAPC and those without HAPC.

Table S4. Differences in basic indicators of different sleep qualities.

Table S5. Differences in fatigue and quality of life indices by sleep quality.

Table S6. Laboratory instrumentation for sample preparation.

Table S7. Brief introduction to the scoring criteria for each clinical scale.

| Table S1. Basic characteristics of 47 Tibetan patients with chronic altitude sickness | | | | |
| --- | --- | --- | --- | --- |
| Variable | Total (N=47) | Sex | | *P* |
|  |  | Males (n=29) | Females (n=18) |  |
| PSQI | 6.00 (4.50, 9.00) | 5.00 (4.00, 8.00) | 8.00 (6.00, 9.75) | 0.048 |
| FS-14 | 7.70 ± 3.10 | 7.00 ± 2.85 | 8.83 ± 3.22 | 0.056 |
| SF-36 |  |  |  |  |
| PF | 80.00 (65.00, 90.00) | 75.00 (65.00, 90.00) | 80.00 (60.00, 90.00) | 0.895 |
| RP | 25.00 (0.00, 75.00) | 0.00 (0.00, 75.00) | 25.00 (0.00, 93.75) | 0.521 |
| RE | 33.00 (0.00, 100.00) | 33.00 (0.00, 100.00) | 33.00 (0.00, 91.75) | 0.898 |
| SF | 62.50 (50.00, 81.25) | 62.50 (50.00, 87.50) | 62.50 (50.00, 75.00) | 0.501 |
| BP | 50.98 ± 17.98 | 53.07 ± 18.36 | 47.61 ± 17.32 | 0.311 |
| MH | 57.19 ± 15.33 | 57.66 ± 14.52 | 56.44 ± 16.96 | 0.803 |
| VT | 61.91 ± 14.50 | 63.62 ± 11.87 | 59.17 ± 18.01 | 0.360 |
| GH | 48.38 ± 16.82 | 49.31 ± 17.18 | 46.89 ± 16.60 | 0.634 |
| Data were expressed as mean ± standard deviation or median (quartiles). Comparisons between groups were made using t-test or Wilcoxon test.Abbreviations: PF (physical functioning), RP (physical problems), RE (role limitations due to emotional problems), SF (social functioning due to emotional problems), BP (bodily pain), MH (mental health), VT (vitality), GH (general health perceptions). | | | | |

| Table S2. Differences in basic indicators between Tibetan patients with HAPC and those without HAPC. | | | |
| --- | --- | --- | --- |
| Variable | High altitude polycythemia | | *P* |
|  | Yes (n=12) | No (n=35) |  |
| Age, years | 42.75 ± 5.15 | 39.83 ± 6.43 | 0.126 |
| Sex |  |  | **0.017** |
| Males | 11 (91.67) | 18 (51.43) |  |
| Females | 1 (8.33) | 17 (48.57) |  |
| CMS |  |  | **0.005** |
| Mild | 0 (0.00) | 2 (5.71) |  |
| Moderate | 1 (8.33) | 19 (54.29) |  |
| Severe | 11 (91.67) | 14 (40.00) |  |
| BMI, kg/m^2^ | 25.51 ± 2.37 | 24.96 ± 2.32 | 0.491 |
| SBP, mmHg | 118.50 (114.75, 132.75) | 114.00 (104.50, 121.50) | **0.049** |
| DBP, mmHg | 85.08 ± 10.28 | 76.06 ± 10.27 | **0.017** |
| RBC, 10^12/L | 7.87 (7.68, 8.06) | 6.01 (5.57, 6.34) | **<0.001** |
| HGB, g/L | 232.83 ± 16.26 | 175.00 ± 20.56 | **<0.001** |
| HCT, % | 72.20 (68.52, 74.88) | 52.40 (48.10, 56.85) | **<0.001** |
| MCV, fL | 89.50 ± 6.75 | 88.55 ± 4.06 | 0.651 |
| MCH, pg | 29.60 (28.18, 31.00) | 29.60 (28.70, 30.90) | 0.751 |
| MCHC, g/L | 328.00 (324.50, 331.00) | 336.00 (332.00, 339.00) | **0.001** |
| RDWcv, % | 14.20 (13.83, 15.93) | 13.80 (13.30, 14.40) | 0.057 |
| RDWsd, fL | 49.90 (47.38, 52.02) | 46.50 (45.10, 47.95) | **0.003** |
| PLT, 10^9/L | 203.00 ± 70.86 | 249.74 ± 69.04 | 0.062 |
| MPV, fL | 10.58 ± 1.04 | 10.30 ± 1.08 | 0.424 |
| PDW, fL | 16.52 ± 0.26 | 16.24 ± 0.40 | **0.010** |
| CHOL, mmol/L | 3.92 (3.57, 4.58) | 3.95 (3.48, 4.61) | 0.951 |
| TG, umol/L | 0.88 (0.74, 1.50) | 1.04 (0.61, 1.33) | 0.836 |
| HDLc, umol/L | 1.04 (0.92, 1.29) | 1.26 (0.99, 1.50) | 0.241 |
| LDLc, umol/L | 2.97 (2.75, 3.66) | 3.15 (2.59, 3.59) | 0.893 |
| ApoA1, g/L | 1.41 ± 0.14 | 1.52 ± 0.18 | 0.050 |
| ApoB, g/L | 0.94 ± 0.40 | 0.91 ± 0.29 | 0.794 |
| BUN, mmol/L | 4.78 ± 1.79 | 4.70 ± 1.42 | 0.893 |
| Cr, mmol/L | 79.73 ± 7.46 | 77.09 ± 14.29 | 0.419 |
| UA, umol/L | 429.13 ± 155.32 | 373.61 ± 96.63 | 0.264 |
| GLU, mmol/L | 4.24 ± 0.65 | 4.76 ± 0.51 | **0.024** |
| T-AOC, U/mL | 19.98 ± 5.87 | 12.99 ± 5.21 | **0.002** |
| CAT, U/mL | 147.00 (33.77, 256.49) | 118.78 (88.32, 143.78) | 0.477 |
| GSH, umol/L | 31.12 (24.93, 35.30) | 21.90 (19.02, 25.94) | **0.014** |
| MDA, umol/L | 4.27 ± 1.69 | 3.29 ± 1.42 | 0.087 |
| SOD, umol/L | 50.36 (48.42, 52.79) | 47.90 (45.65, 51.56) | 0.246 |
| 8-OHdG, ng/mL | 28.26 (21.12, 37.22) | 22.56 (15.50, 37.35) | 0.367 |
| Data were expressed as mean ± standard deviation, median (quartiles), or n (percentage). Comparisons between groups were made using t-test or Wilcoxon test. Abbreviations: HAPC (high altitude polycythemia; Yes/No), CMS (chronic mountain sickness; Mild/Moderate/Severe), BMI (body mass index), SBP/DBP (systolic/diastolic blood pressure), RBC (red blood cells), HGB (hemoglobin), HCT (hematocrit), MCV (mean corpuscular volume), MCH (mean corpuscular hemoglobin), MCHC (MCH concentration), RDWcv/RDWsd (red cell distribution width, coefficient of variation/standard deviation), PLT (platelet count), MPV (mean platelet volume), PDW (platelet distribution width), CHOL (total cholesterol), TG (triglycerides), HDLc/LDLc (high/low-density lipoprotein cholesterol), ApoA1/ApoB (apolipoprotein A1/B), BUN (blood urea nitrogen), Cr (creatinine), UA (uric acid), GLU (glucose), T-AOC (total antioxidant capacity), CAT (catalase), GSH (glutathione), MDA (malondialdehyde), SOD (superoxide dismutase), and 8-OHdG (8-hydroxy-2’-deoxyguanosine). | | | |

| Table S3. Differences in sleep, fatigue, and quality of life between Tibetan patients with HAPC and those without HAPC. | | | |
| --- | --- | --- | --- |
| Variable | High altitude polycythemia | | *P* |
|  | Yes (n=12) | No (n=35) |  |
| PSQI | 5.00 (4.00, 7.25) | 8.00 (5.00, 9.50) | 0.147 |
| FS-14 | 7.00 ± 3.02 | 7.94 ± 3.13 | 0.366 |
| SF-36 |  |  |  |
| PF | 75.00 (63.75, 91.25) | 80.00 (70.00, 90.00) | 0.439 |
| RP | 12.50 (0.00, 75.00) | 25.00 (0.00, 87.50) | 0.697 |
| RE | 16.50 (0.00, 100.00) | 33.00 (0.00, 100.00) | 0.866 |
| SF | 68.75 (62.50, 87.50) | 62.50 (50.00, 75.00) | 0.082 |
| BP | 51.50 ± 20.18 | 50.80 ± 17.48 | 0.916 |
| MH | 57.67 ± 16.22 | 57.03 ± 15.26 | 0.906 |
| VT | 62.50 ± 13.73 | 61.71 ± 14.95 | 0.869 |
| GH | 48.17 ± 19.81 | 48.46 ± 16.00 | 0.964 |
| Data were expressed as mean ± standard deviation or median (quartiles). Comparisons between groups were made using t-test or Wilcoxon test. Abbreviations: PF (physical functioning), RP (physical problems), RE (role limitations due to emotional problems), SF (social functioning due to emotional problems), BP (bodily pain), MH (mental health), VT (vitality), GH (general health perceptions). | | | |

| Table S4. Differences in basic indicators of different sleep qualities. | | | |
| --- | --- | --- | --- |
| Variable | PSQI | | *P* |
|  | PSQI > 7 (n=21) | PSQI ≤ 7(n=26) |  |
| Age, years | 40.33 ± 7.01 | 40.77 ± 5.62 | 0.818 |
| Sex |  |  | 0.037 |
| Males | 9 (42.86) | 20 (76.92) |  |
| Females | 12 (57.14) | 6 (23.08) |  |
| CMS |  |  | 0.481 |
| Mild | 1 (4.76) | 1 (3.85) |  |
| Moderate | 11 (52.38) | 9 (34.62) |  |
| Severe | 9 (42.86) | 16 (61.54) |  |
| BMI, kg/m^2^ | 24.58 ± 2.46 | 25.51 ± 2.15 | 0.180 |
| SBP, mmHg | 115.00 (106.00, 122.00) | 116.00 (106.25, 126.50) | 0.607 |
| DBP, mmHg | 76.38 ± 9.26 | 79.96 ± 12.02 | 0.255 |
| RBC, 10^12/L | 5.90 (5.56, 6.28) | 6.56 (6.04, 7.56) | **0.015** |
| HGB, g/L | 179.05 ± 29.99 | 198.42 ± 31.51 | **0.037** |
| HCT, % | 51.20 (47.80, 57.80) | 57.75 (54.50, 66.85) | **0.023** |
| MCV, fL | 88.66 ± 4.77 | 88.90 ± 4.95 | 0.867 |
| MCH, pg | 29.60 (28.50, 31.00) | 29.65 (28.60, 30.78) | 0.889 |
| MCHC, g/L | 332.00 (329.00, 338.00) | 334.00 (329.00, 338.50) | 0.974 |
| RDWcv, % | 13.90 (13.40, 14.60) | 13.80 (13.33, 14.47) | 0.772 |
| RDWsd, fL | 47.80 (46.00, 49.00) | 46.55 (45.23, 48.95) | 0.398 |
| PLT, 10^9/L | 248.86 ± 81.95 | 228.88 ± 62.56 | 0.363 |
| MPV, fL | 10.21 ± 1.10 | 10.50 ± 1.04 | 0.377 |
| PDW, fL | 16.19 ± 0.43 | 16.42 ± 0.33 | 0.054 |
| CHOL, mmol/L | 3.97 (3.52, 4.43) | 3.95 (3.51, 4.59) | 0.906 |
| TG, umol/L | 0.78 (0.62, 1.06) | 1.19 (0.78, 1.42) | 0.104 |
| HDLc, umol/L | 1.41 (1.05, 1.58) | 1.10 (0.92, 1.27) | **0.025** |
| LDLc, umol/L | 2.83 (2.51, 3.24) | 3.21 (2.87, 3.92) | 0.129 |
| ApoA1, g/L | 1.57 ± 0.19 | 1.42 ± 0.14 | **0.006** |
| ApoB, g/L | 0.85 ± 0.27 | 0.98 ± 0.34 | 0.147 |
| BUN, mmol/L | 357.73 ± 84.90 | 412.05 ± 131.45 | 0.094 |
| Cr, mmol/L | 4.67 ± 0.55 | 4.59 ± 0.63 | 0.664 |
| UA, umol/L | 1.57 ± 0.19 | 1.42 ± 0.14 | **0.006** |
| GLU, mmol/L | 0.85 ± 0.27 | 0.98 ± 0.34 | 0.147 |
| T-AOC, U/mL | 13.70 ± 6.83 | 15.63 ± 5.52 | 0.301 |
| CAT, U/mL | 120.80 (94.20, 188.95) | 121.08 (71.30, 143.44) | 0.156 |
| GSH, umol/L | 22.48 (20.17, 28.24) | 23.34 (20.32, 34.58) | 0.668 |
| MDA, umol/L | 3.32 ± 1.51 | 3.72 ± 1.57 | 0.378 |
| SOD, umol/L | 48.04 (47.15, 51.33) | 48.34 (45.65, 52.60) | 0.724 |
| 8-OHdG, ng/mL | 21.07 (14.13, 23.61) | 32.40 (20.73, 40.12) | **0.024** |
| Data were expressed as mean ± standard deviation, median (quartiles), or n (percentage). Comparisons between groups were made using t-test or Wilcoxon test. Abbreviations: HAPC (high altitude polycythemia; Yes/No), CMS (chronic mountain sickness; Mild/Moderate/Severe), BMI (body mass index), SBP/DBP (systolic/diastolic blood pressure), RBC (red blood cells), HGB (hemoglobin), HCT (hematocrit), MCV (mean corpuscular volume), MCH (mean corpuscular hemoglobin), MCHC (MCH concentration), RDWcv/RDWsd (red cell distribution width, coefficient of variation/standard deviation), PLT (platelet count), MPV (mean platelet volume), PDW (platelet distribution width), CHOL (total cholesterol), TG (triglycerides), HDLc/LDLc (high/low-density lipoprotein cholesterol), ApoA1/ApoB (apolipoprotein A1/B), BUN (blood urea nitrogen), Cr (creatinine), UA (uric acid), GLU (glucose), T-AOC (total antioxidant capacity), CAT (catalase), GSH (glutathione), MDA (malondialdehyde), SOD (superoxide dismutase), and 8-OHdG (8-hydroxy-2’-deoxyguanosine). | | | |

| Table S5. Differences in fatigue and quality of life indices by sleep quality. | | | |
| --- | --- | --- | --- |
| Variable | PSQI | | *P* |
|  | PSQI > 7(n=21) | PSQI ≤ 7 (n=26) |  |
| FS-14 | 8.86 ± 2.57 | 6.77 ± 3.22 | **0.017** |
| SF-36 |  |  |  |
| PF | 75.00 (60.00, 80.00) | 85.00 (66.25, 95.00) | 0.182 |
| RP | 25.00 (0.00, 75.00) | 25.00 (0.00, 75.00) | 0.837 |
| RE | 0.00 (0.00, 67.00) | 67.00 (0.00, 100.00) | **0.018** |
| SF | 62.50 (50.00, 75.00) | 62.50 (50.00, 84.38) | 0.622 |
| BP | 42.00 ± 17.72 | 58.23 ± 14.87 | **0.002** |
| MH | 54.48 ± 15.87 | 59.38 ± 14.82 | 0.284 |
| VT | 57.86 ± 16.48 | 65.19 ± 12.04 | 0.097 |
| GH | 41.52 ± 14.34 | 53.92 ± 16.87 | **0.009** |
| Data were expressed as mean ± standard deviation or median (quartiles). Comparisons between groups were made using t-test or Wilcoxon test. Abbreviations: PF (physical functioning), RP (physical problems), RE (role limitations due to emotional problems), SF (social functioning due to emotional problems), BP (bodily pain), MH (mental health), VT (vitality), GH (general health perceptions). | | | |

| Table S6. Laboratory instrumentation for sample preparation. | | |
| --- | --- | --- |
| **Equipment Name** | **Manufacturer** | **Location** |
| AUY120 analytical balance | Shimadzu | Kyoto, Japan |
| D3024 Benchtop High-Speed Micro Centrifuge | SCILOGEX | Rocky Hill, CT, USA |
| D3024R Benchtop High-Speed Refrigerated Micro Centrifuge | SCILOGEX | Rocky Hill, CT, USA |
| MX-S Vortex Mixer | SCILOGEX | Rocky Hill, CT, USA |
| KZ-III-F High-throughput Low-temperature Tissue Grinder | Servicebio | Wuhan, China |
| SLK-O3000-S Digital Control Orbital Shaker | SCILOGEX | Rocky Hill, CT, USA |
| THZ-82A Digital Display Thermostatic Air Bath Shaker | Langyue Instrument | Changshu, China |
| TL-420D Water Bath | Tianli Medical Instrument | Taizhou, China |
| HD500L-2R0 system | Haideneng Fluid Engineering | Qingdao, China |
| BCD-190TMPK refrigerator | Haier | Qingdao, China |

| Table S7. Brief introduction to the scoring criteria for each clinical scale. | | | | |
| --- | --- | --- | --- | --- |
| **Scale Name** | **Score Range** | **Scoring Method** | **Score Interpretation** | **Key Notes** |
| **PSQI** | **Total Score: 0-21** | **Sum of 7 component scores:** Subjective sleep quality, Sleep latency, sleep duration, Habitual sleep efficiency, Sleep disturbances, Use of sleep medication, Daytime dysfunction. Subjective sleep quality scored 1-3; the other six components scored 0-3. | **Higher total score indicates worse sleep quality.** For Chinese populations: **> 7** identifies individuals with poor sleep quality (Sensitivity: 98.3%, Specificity: 90.2%). | Calculate based on component definitions and scoring methods. **Example (Sleep efficiency):** = (Actual sleep duration / Time in bed) × 100% → then converted to a 0-3 score based on percentage. |
| **FS-14** | **Total Score: 0-14** | **Binary scoring:** “Yes” = 1 point, “No” = 0 point. **Reverse-scored items:** Item 10: “Is your mind as clear and agile as usual when thinking?” (“Yes” = 0, “No” = 1); Item 13: “Is your memory as good as usual now?” (“Yes” = 0, “No” = 1); Item 14: “Do you still enjoy doing the habitual things you used to do?” (“Yes” = 0, “No” = 1) | **Higher total score indicates greater fatigue severity.** | **Reverse-scored items must be noted.** |
| **SF-36** | **Per Dimension: 0-100** | **Calculated across 8 dimensions:** Physical Functioning (PF), Role-Physical (RP), Bodily Pain (BP), General Health (GH), Vitality (VT), Social Functioning (SF), Role-Emotional (RE), Mental Health (MH). **Transformation formula:** Dimension Score = [(Actual Raw Score - Theoretical Minimum Score) / (Theoretical Maximum Score - Theoretical Minimum Score)] × 100 | **Higher dimension score indicates better health status.** | **Raw scores cannot be summed directly.** **Example (PF transformation):** [(Actual Raw Score - 10) / 20] × 100 |
